# Bridging Disciplines: Comprehensive Approaches to Cardiovascular Disease Treatment

**DOI:** 10.3390/life15030479

**Published:** 2025-03-17

**Authors:** Daniela Maria Tanase, Anca Victorita Trifan, Mariana Floria

**Affiliations:** 1 Department of Internal Medicine, “Grigore T. Popa” University of Medicine and Pharmacy, 700111 Iasi, Romania; anca.trifan@umfiasi.ro (A.V.T.); floria.mariana@umfiasi.ro (M.F.); 2 Clinic of Internal Medicine, “St. Spiridon” Emergency Hospital Iasi, 700111 Iasi, Romania; 3 Institute of Gastroenterology and Hepatology, “St. Spiridon” Emergency Hospital Iasi, 700111 Iasi, Romania

Cardiovascular disease (CVD) remains one of the leading causes of death worldwide. The human body is a remarkable structure composed of individual organs that also function as a complex system with multiple physiological matrices that interact continuously and influence each other. The perplexing association between cardiovascular disease and non-cardiovascular disease is bound by common risk factors that contribute not only to disease onset but also various other intricate complications. The liver–heart, bowel–heart, brain–heart, and kidney–heart axes are some of the areas interdisciplinary teams focus on in order to elucidate the gamut of pathophysiologic interferences, help prevent them, and find novel diagnosis algorithms and the best therapeutic approaches for these patients.

Besides the commonly known risk factors, it is important to emphasize the direct impacts of work and psychosocial stress, environmental health, and socioeconomic status on CVD development. Poor adherence to therapy also contributes to negative outcomes and constitutes a major health problem. Health-related quality of life (HRQoL) has been used as a secondary or co-primary endpoint in studies and has been inversely associated with multimorbidity; however, it is understudied in lower-income countries, and the poor quality of various HRQoL instruments can threaten measurement accuracy in health-related economic evaluations and clinical studies [1].

Florescu et al. [2] investigated HRQoL assessment in relation to patients with CVD and non-conventional CVD risk factors (CRFs) using the WHOQOL-BREF questionnaire. They showed that subjects with CRFs and requiring more than one drug had lower self-reported HRQoL in relation to psychological and physical health. They noted the negative impact of arterial hypertension on HRQoL and that treatment adherence had an inversely proportional relationship with the age of the patients included. The results showed that subjects with CRFs were more sedentary (*p* = 0.001), with higher BMI values (*p* < 0.001) than the healthy subjects, and that those taking cardiology medication were older and had lower values regarding social relationships. This is one of the few studies assessing the interplay between the self-reported HRQoL of working young and middle-aged adults, the need for CV treatment, and the presence of CRFs. This study also underscores the crucial interaction between psychological, socioeconomic, environmental, and somatic health. Importantly, one of the limitations of this study was the similarity in sex, age, distribution, degree of education, and socio-economic class between those with CVD and the controls.

In this regard, the American College of Cardiology and American Heart Association also highlights the importance of individual-level social determinants of health (e.g., race, ethnicity, sex, gender identity, immigration, and acculturation), as well as those corresponding to the interpersonal (disparate healthcare quality, individual education and income, diet quality, and food insecurity), community, and societal levels (racial and ethnic segregation, health care services, and research infrastructure), on HRQoL assessments and overall results [3].

The need for better quality of care has directed attention to the measurement and reporting of processes of care and patient outcomes. The European Society of Cardiology (ESC) also stresses the importance of HRQoL assessment and has developed quality indicators (QIs) to evaluate the management of a range of common cardiovascular diseases based on a standardized methodology [4]. Experts have proposed the HeartQoL questionnaire, which was validated in each of the three major ischemic heart disease diagnoses, namely, angina, myocardial infarction, and heart failure, as a suitable instrument for health-related- and CVD quality-of-life assessment [5,6].

Within cardiovascular disease, calcific aortic stenosis is one of the most relevant vascular heart diseases (VHDs), affecting almost 9 million people globally and being linked with a poor prognosis if left untreated. The increased prevalence of non-rheumatic aortic VHD comes in parallel with an increasingly aging population, and its consequences are marked by hemodynamic obstructions of left-ventricular outflow. The medication approach to addressing cardiovascular disease has some limitations in specific conditions, such as aortic stenosis (AS). Although dyslipidemia, the major contributor to VHD, is being targeted by not only cardiologists but also other specialists, the “curative” treatment to date is the surgical replacement of the aortic valve with an artificial mechanical or biological prosthetic valve [7]. In the last decade, attention has been paid to new prevention methods, diagnostic tools, and therapeutic options.

Regarding VHD diagnosis, a recent review by Lisi et al. [8] showed the importance of the early detection of left-ventricle and left-atrium remodeling in AS, before and after aortic valve replacement via speckle-tracking echocardiography (STE), an easy, fast, and advanced tool allowing early detection of the functional and structural characteristics of the myocardium. STE helps measure peak atrial longitudinal strain (PALS), which is superior to conventional echocardiographic parameters for the diagnostic and prognostic evaluation of heart failure with both a reduced and preserved ejection fraction, constituting a strong predictor of pre-and postoperative atrial arrhythmia, and it may help distinguish between patients with pre- and post-capillary pulmonary hypertension.

Although not included in the current guidelines as an initial evaluation for these patients since it requires dedicated acquisition and is time-consuming, researchers recommend including STE/global longitudinal strain (GLS) in VHD before and after valve implantation procedures. For patients with symptomatic severe aortic stenosis who have undergone TAVI, the use of STE can aid in the risk stratification of asymptomatic patients in order to determine the optimal timing of valve replacement [9]. Thus, its routine use in clinical practice could improve earlier surgical interventions and promote better myocardial remodeling and outcomes for these patients.

Severe AS treatment involves surgical or transcatheter aortic valve replacement. SAVR remained the gold standard option for many years for symptomatic individuals with severe AS; however, TAVI/TAVR has surpassed it, with evidence showing its superiority as a first-choice therapy for AS in elderly patients. Bioprosthetic valves used for SAVR or TAVR have limited durability due to structural valve deterioration (SVD) found at echocardiographic follow-ups. SVD definitions include hemodynamic and structural changes and are associated with a raised risk of rehospitalization due to heart failure and a trend towards a higher risk of cardiovascular mortality. Transcatheter prostheses may exhibit better systolic hemodynamic performance than surgical bioprostheses. The physiopathological mechanisms behind the valve’s deterioration are multifactorial; prosthesis durability is dependent on the model used and an individual’s characteristics and comorbidities [10].

The gold standard for prosthesis preparation remains the glutaraldehyde (GA) fixation of bovine pericardium, which adversely promotes calcification and requires a secondary re-operation after 10–15 years. Calcification is mostly caused by the intercalation of calcium hydroxyapatite in pericardium collagen bundles. To improve heart valve bioprostheses outcomes and ameliorate SVD, researchers are attempting to develop a better structural characterization of bioprosthesis materials. In their original research, Welzel et al. [11] demonstrated that GA-free preparation strategies might improve the lifespans of bioprostheses, simultaneously crosslinking collagen fibers, lowering immunogenicity, and making the material sterile. The use of the novel GA-free stabilization method and the sterilization of acellular pericardial scaffolds by combining photo-initiated ultraviolet cross-linking with low-energy electron irradiation (SULEEI) treatment of bovine pericardium improves the lifespan of bioprostheses as it is rich in the collagen-typical α-helical substructure, which preserves proteins’ secondary structures, making them less prone to deterioration. Additionally, in the three-step SULEEI protocol, bovine pericardium is first decellularized to remove DNA and cellular material, preserving the extracellular matrix (ECM). These results, found via IR spectroscopy, emphasize the beneficial effects of SULEEI preparation, as a promising alternative for application in cardiac surgery due to preserving the typical α-helical substructure of collagen in bovine pericardium, yielding superior results compared to the use of GA-fixed tissue samples.

On another note, many patients with valvular disease or any other cardiovascular disease are characterized to some degree by a chronic, non-resolving inflammatory atherosclerotic (ATS) pattern. Systemic vascular impairment is exacerbated by oxidative stress, cells of the innate immune system, and pro-inflammatory cell activation, which prolong ATS plaque development and increase the risk of thrombosis, coronary artery disease (CAD), and, subsequently, CVD complications such as acute cardiac ischemia or stroke. Given the high rates of stroke—the second leading cause of mortality worldwide—and the impact of CVD on the onset of cerebral vascular chronic or acute disease, research into the brain–heart axes might be able to explain the pathophysiologic interferences behind these two systems and thus provide us with a comprehensive understanding of the clinical cardiovascular consequences. Current guidelines draw attention to the relevance of the pre-test clinical likelihood of obstructive atherosclerotic coronary artery disease. Laboratory blood tests may identify potential causes of ischemia and cardiovascular risk factors and can yield prognostic information [12]. Although blood tests—namely, the acquisition of lipid profiles, a full blood count, renal function analysis, determination of glycemic status along with HbA1c and/or fasting plasma glucose, the assessment of thyroid function (at least once), and the determination of hs-CRP and/or fibrinogen plasma levels—are recommended for all individuals to refine risk stratification, diagnose comorbidities, and guide treatment, their value is limited beyond identifying traditional risk factors.

CAD severity is associated with prevalent systemic pro-inflammatory polarization. Specifically, neutrophils are present in the arterial intima and the atherosclerotic plaques and can promote atherosclerotic plaque instability. As part of the SMARTool European Project (NCT04448691), Sbrana et al. [13] investigated the roles of blood neutrophil quantities and phenotypes and lipid-rich necrotic core volume (LRNCV) in 55 stable CAD individuals. Coronary plaques were detected via computed tomography coronary angiography (CTCA). First, they found that the overall white blood cell (WBC) count (n◦/µL) and the neutrophil cell count (n◦/µL) were positively linked with the LRNCV values according to multiple regression. Also, neutrophil CD11b expression (as RFI) correlated positively with IL-6 plasma levels (*p* = 0.0043; R = 0.379) and inversely with the IL-10/IL-6 ratio (*p* = 0.0099; R = 0.345). Upregulation of integrin/activation membrane neutrophil marker CD11b expression increases respiratory burst activity of phagocytes and can enhance plaque instability. Second, the authors demonstrated that an increased capacity for neutrophil–platelet mutual interaction, expressed via the Neutrophil–PLT adhesion index, was significantly associated with the LRNCV values according to multiple regression.

This study provides further insights into the pathophysiological mechanisms of atherosclerosis and the importance of blood neutrophils’ phenotypes as potential biomarkers or therapeutic targets, as an even longer long-lasting activation status of circulating neutrophils contributes to the development, evolution, and possible onset of acute ischemic events in stable CAD patients.

Another two ATS complications mentioned are stroke and transient ischemic attack. Despite the current guidelines that focus on interventions that include a multidisciplinary-team-based approach to care for risk factor control, treatment, and secondary prevention after an ischemic stroke, early diagnostic assessment and tools should must also be considered [14]. Interestingly, in their retrospective pilot study, Aftyka et al. [15] demonstrated that heart rate variability (HRV) might predict which hemisphere of the brain is affected during an acute ischemic stroke (AIS) with a right hemispheric (RH) or left hemispheric (LH) lesion. Their study included 64 patients who suffered an AIS (25 and 39 who suffered RH and LH strokes, respectively) analyzed using the 24 h Holter ECG records at *NN* intervals performed at a mean of 4.3 ± 2 days following their AIS using linear and non-linear methods. They found decreased HRV complexity in the right-hemispheric-stroke patients (lower sample entropy) associated with a higher ratio of heart failure (most likely due to higher activity in the sympathetic nervous system) compared to the LH subjects, suggesting that HRV could be useful in the discrimination of hemispheric involvement in AIS. These results are supported by other studies showing that impaired cardiac autonomic function, quantified by employing heart rate variability (HRV) measured via ECG recording, seems to be an independent predictor of stroke or systemic embolism and that point out the relevance of heart rate during the acute period of ischemic stroke as a predictor of major clinical events. Optimal heart rate control might be a target for preventing subsequent CVD events [16]. However, when we interpret these studies’ results, we must consider that, frequently, subjects with increased heart rate may have comorbid conditions (sepsis, hyperthyroidism, or even ventricular dysfunction) or that their condition may be a marker of elevated sympathetic activity secondary to stroke.

As observed, researchers are bringing new data on cardiovascular disease to the fore, with topics ranging from novel biomarkers and risk assessment to diagnostic strategies. This vast field, which encompasses integrative practices, has the potential to allow the gathering of research teams from different specialties united by cardiovascular disease as a main bridge. Newer research is expected, which may help establish heart team guidelines for better disease prevention and outcomes.
